# Dealing with uncertain results from chromosomal microarray and exome sequencing in the prenatal setting: An international cross‐sectional study with healthcare professionals

**DOI:** 10.1002/pd.5932

**Published:** 2021-03-30

**Authors:** Celine Lewis, Jennifer Hammond, Jasmijn E. Klapwijk, Eleanor Harding, Stina Lou, Ida Vogel, Emma J. Szepe, Lisa Hui, Charlotta Ingvoldstad‐Malmgren, Maria J. Soller, Kelly E. Ormond, Mahesh Choolani, Melissa Hill, Sam Riedijk

**Affiliations:** ^1^ Population, Policy and Practice UCL Great Ormond Street Institute of Child Health London UK; ^2^ North Thames Genomic Laboratory Hub Great Ormond Street Hospital London UK; ^3^ Genetic and Genomic Medicine UCL Great Ormond Street Institute of Child Health London UK; ^4^ Department of Clinical Genetics Erasmus MC Rotterdam The Netherlands; ^5^ BSc Paediatrics and Child Health The UCL Great Ormond Street Institute of Child Health London UK; ^6^ Center for Fetal Diagnostics Aarhus University Hospital Aarhus Denmark; ^7^ Reproductive Epidemiology Murdoch Children's Research Institute Royal Children's Hospital Parkville Victoria Australia; ^8^ Department of Paediatrics Faculty of Medicine Dentistry and Health Sciences University of Melbourne Melbourne Victoria Australia; ^9^ Department of Obstetrics and Gynaecology University of Melbourne Parkville Victoria Australia; ^10^ Department of Perinatal Medicine Mercy Hospital for Women Heidelberg Victoria Australia; ^11^ Department of Obstetrics and Gynaecology Northern Health Epping Victoria Australia; ^12^ Center for Fetal Medicine Karolinska University Hospital Stockholm Sweden; ^13^ Department of Clinical Genetics Karolinska Hospital and Department of Molecular Medicine and Surgery Karolinska Institutet Stockholm Sweden; ^14^ Department of Genetics and Stanford Center for Biomedical Ethics Stanford University School of Medicine Stanford California USA; ^15^ Department of Obstetrics & Gynaecology National University Hospital Singapore; ^16^ Yong Loo Lin School of Medicine National University of Singapore Singapore

## Abstract

**Objectives:**

To conduct qualitative interviews with healthcare providers working in different countries to understand their experiences of dealing with uncertain results from prenatal chromosome microarray analysis (CMA) and exome sequencing (ES).

**Methods:**

Semi‐structured interviews with 31 healthcare providers who report or return prenatal CMA and/or ES results (clinicians, genetic counsellors and clinical scientists) in six countries with differing healthcare systems; Australia (4), Denmark (5), Netherlands (6), Singapore (4), Sweden (6) and United Kingdom (6). The topic guide explored the main sources of uncertainty and their management.

**Results:**

There was variation in reporting practices both between and across countries for variants of uncertain significance, however, there was broad agreement on reporting practices for incidental findings. There was also variation in who decides what results are reported (clinical scientists or clinicians). Technical limitations and lack of knowledge (to classify variants and of prenatal phenotypes) were significant challenges, as were turnaround times and lack of guidelines.

**Conclusion:**

Health professionals around the globe are dealing with similar sources of uncertainty, but managing them in different ways, Continued dialogue with international colleagues on ways of managing uncertain results is important to compare and contrast the benefits and limitations of the different approaches.

## Key points

What's already known about this topic?

- Chromosome microarray analysis and exome sequencing have increased diagnostic yield over karyotyping but have increased the incidence of uncertain results.

What does this study add?

- Our findings highlight variation in reporting practices both between and across countries for variants of uncertain significance, although there is broad agreement on reporting practices for incidental findings.
- International guidelines may help to standardise how we define and categorise variants, however, global uniformity on the management of uncertain results may not be a realistic or desirable goal.

## 1 INTRODUCTION

Routine fetal ultrasound identifies structural abnormalities in around 3% of pregnancies.^1^ Traditionally, investigations to identify the underlying aetiology have relied on cytogenetic analysis, including karyotyping. Over the last 10 years, chromosome microarray analysis (CMA) has been widely adopted as the first‐line test to detect submicroscopic pathogenic copy number variations, and has been shown to increase diagnostic yield over traditional karyotyping.^2^ Genome sequencing (primarily through whole exome sequencing [ES] and targeted panels) is now increasingly being used in the prenatal setting, and has increased the frequency with which genetic causes are detected with karyotyping or CMA alone,^3^, ^4^ impacting clinical management through informing decisions around continuation of pregnancy.^5^

A key counselling and ethical challenge associated with CMA and ES is the potential to detect variants of uncertain significance (VUS) or incidental findings (IFs) (findings additional to the original reason for testing which are *not* actively searched for) which may have implications for both the fetus and the parent(s).^6^ Although uncertainty in the prenatal setting is not new, the scale and types of uncertainty that may occur is increasing because of the more detailed comprehensive analysis of the fetal genome. For example, detection of VUS has been reported in around 2%–6% of prenatal cases through CMA^2^, ^7^, ^8^, ^9^ and around 4%–20% of cases through ES,^3^, ^10^, ^11^ although the proportion is likely to decrease as new knowledge is gained and VUS are reanalysed.^11^

A body of evidence has been published over recent years looking at healthcare professionals (HPs) experiences and attitudes towards returning uncertain results following prenatal CMA, and more recently ES. These studies have been conducted in countries including the Netherlands,^12^ United States of America (USA),^13^, ^14^, ^15^ United Kingdom (UK),^16^, ^17^, ^18^ Hong Kong^19^ and Australia.^20^, ^21^ The findings have highlighted differences in opinions and practice both across and within countries, regarding the management of uncertain results.^22^ This is not surprising given the lack of consensus from some of the most notable professional bodies in terms of specific guidance around the reporting of uncertain results.^23^, ^24^, ^25^, ^26^, ^27^, ^28^, ^29^ Here we report the findings from an international cross‐sectional study of multidisciplinary HPs involved in prenatal diagnosis. The aims of the study were to (1) identify the different sources of uncertainties that HPs are regularly experiencing as a result of prenatal CMA and/or ES, and (2) describe how these are being managed.

## 2 METHODS

### 2.1 Ethical approval

Local ethical approval was gained from each participating research team (see Supporting Information Ethical Approval).

### 2.2 Study design

This was a qualitative study using purposive sampling (the identification and selection of participants who are especially knowledgeable about or experienced with the phenomenon of interest) and semi‐structured interviews with (1) clinical scientists—sometimes referred to as laboratory scientists—who analyse and report prenatal CMA and/or ES results, and (2) clinicians who conduct posttest counselling around prenatal CMA and/or ES (e.g., geneticists, genetic counsellors, obstetricians, maternal fetal medicine specialists). We focused on clinicians who conduct posttest counselling as they are most likely to have an in‐depth understanding of what types of uncertain results get reported and how such results are managed. However, some also had experience of conducting pretest counselling.

### 2.3 Study setting

Participants from six countries took part in interviews using the same topic guides: Australia, Denmark, The Netherlands, Singapore, Sweden, and the UK. These high‐income countries were chosen as they have established prenatal screening and diagnosis programs within a range of healthcare systems, and previous research has also highlighted that health professionals from some of these countries hold differing attitudes towards prenatal testing.^30^

At the time of the interviews, prenatal CMA and ES (where it was available clinically) were provided as part of a national health service or in an insurance‐based system in Denmark, the Netherlands, Sweden. In the UK, CMA was available as part of prenatal testing in the National Health Service, but ES was only available in research settings pending national implementation. In Australia, CMA was covered by the national health service or an insurance‐based system, but ES was self‐funded. In Singapore, CMA was subsidised in public hospitals but self‐funded in private hospitals, and ES was entirely self‐funded.

### 2.4 Theoretical framework

The overarching theoretical framework that guided the research was Han's taxonomy of uncertainty whereby uncertainty in healthcare is characterised according to its fundamental *sources* (cause of uncertainty), *issues* (implications of uncertainty) and *locus* (with whom the uncertainty lies).^31^ In particular, we referred to a taxonomy of medical uncertainties that related to clinical next‐generation sequencing.^32^ This was developed by Han et al. to facilitate recognition of the uncertainties inherent in each step of genomic testing and help researchers, clinicians, patients and relatives establish realistic expectations of its processes and outcomes. Whilst Han's taxonomy informed the development of the topic guides and our analysis, the aim of this research was not to develop a definitive list of uncertainties in prenatal genomics (this has largely been addressed already^32^), but rather to gain a ‘snapshot' of which uncertainties are frequently encountered by clinical scientists and clinicians in their day‐to‐day practice (i.e., to explore *sources* and *locus*), the associated challenges, and how they are managed (*issues*) across different countries.

### 2.5 Recruitment

Potential participants who were experts in the field and encompassed either laboratory diagnostics or face‐to‐face clinical care were identified and invited to take part in an interview via email, telephone or face‐to‐face. This approach was taken due to the small sample of experts working in this area, most of whom are known to the research team in each participating country. The recruitment target in each country was approximately five interviews including a mix of clinical scientists and clinicians to ensure that both the clinical and laboratory viewpoints were included. Participants could be recruited from one or multiple sites. Informed consent was obtained from all participants.

### 2.6 Interviews

The development of the topic guide was informed by our theoretical framework, the literature^14^, ^15^, ^20^ and was revised following feedback from the authors. The topic guide explored the main *sources* of uncertainty from CMA/ES and how these uncertainties are *managed*. Clinical scientists were asked a question relating to variant classification protocols that are used in the laboratory and clinicians were asked questions on pre‐ and posttesting counselling experiences (see Supporting Information Topic guides). In most cases, questions referred to both CMA/ES together, although some questions asked participants about their views and/or experience of CMA and ES individually.

The topic guides were translated (where necessary) by the co‐authors who are bilingual and work in two languages in their daily professional capacity. Semi‐structured interviews were conducted by the co‐authors in their respective countries (E. J. Szepe, S. Lou, J. Klapwijk, C.Ingvoldstad‐Malmgren, C. Lewis) other than in Singapore (C. Lewis). Interviews were conducted in the participant's native language and then translated into English by the co‐authors, other than in Sweden where the interviewer chose to conduct interviews in English as all the participants spoke English fluently. Interviews were digitally recorded, transcribed verbatim and anonymised. The exception was one interview conducted in Denmark, where the participant preferred to receive the questions by email and respond in writing.

### 2.7 Data analysis

Data were analysed using thematic analysis^33^ using a ‘codebook approach’^34^ whereby questions in the topic guide initially informed potential codes of interest but where additional codes were identified inductively from the data. Data were coded by C. Lewis and J. Hammond and multiple iterations of the codebook were developed. Codes were grouped into subthemes, for example, eligibility for testing, and themes for example, managing uncertainty. These were informed through the theoretical framework, but also through discussion with the authors about how the themes and subthemes logically fitted together to tell a story about uncertainty in prenatal genomics. NVivo version 12 (QSR International, Pty Ltd) was used to facilitate the initial coding and data analysis. We also employed a framework approach, whereby data was copied into an excel spreadsheet in order to conduct structured comparisons between countries for certain questions, for example, to compare approaches to returning VUS.^35^

## 3 FINDINGS

In total, 31 participants from 14 hospital sites agreed to take part (79% recruitment rate) between January 2019 and March 2020. Twenty interviews were conducted face‐to‐face, ten were over telephone (range: 22–82 min; mean = 47 min) and one was via email. Whilst this particular interview was conducted in a different manner to the other 20, it was included on the basis that it was complementary to the verbal interviews and yielded information of relevance to the research question.

### 3.1 Participant characteristics

Table 1 provides a breakdown of participant characteristics. At the time of interview, all participants had experience of reporting or returning prenatal CMA results, but only participants from Australia, Denmark, the Netherlands and Sweden had experience of reporting or returning prenatal ES results during pregnancy. In the UK, ES was being conducted as part of research studies.^3^, ^36^

**TABLE 1:** Participant characteristics

Participant characteristics	*N* = 31
Age	Range: 30–64 years, mode = 45 years
Years in profession	Range: 5–30, mode = 15 years
Gender	
Female	24
Male	7
Interviews per country^a^	
The Netherlands	6
Sweden	6
UK	6
Denmark	5
Australia	4
Singapore	4
Professional background	
Clinical geneticist	12
Clinical scientist	11
Obstetrician	4
Genetic counsellor	2
Fetal medicine consultant	1
Paediatrician	1
Hospital type	
Public	29
Public and private	2

Abbreviations: CMA, chromosome microarray analysis; ES, exome sequencing.

^a^ Participants from Australia were recruited through two sites, the Melbourne Academic Centre for Health Women's and Newborn Health Network, and are representative of Australian practitioners working in publicly funded metropolitan health services. Participants from Denmark were recruited from all the three genetic centres where prenatal samples are analysed. There is a publicly funded national screening program in Denmark resulting in relatively uniform services being provided, however new methods (CMA or ES) have been implemented prenatally at different timepoints between the three centres. Participants from the Netherlands were recruited from one of the eight academic hospitals in the Netherlands that provides prenatal genetic testing. Participants from Singapore were recruited from two out of six sites across the country where prenatal CMA and ES are performed, and are representative of Singaporean practitioners working in government funded health services. Participants from Sweden were recruited from four out of six sites across the country where prenatal CMA and ES are performed and where there is both a genetic and a specialist obstetrician taking care of the patients. Participants from the UK were recruited from one regional genetics service in London (of which there are 21). Participants in the UK were recruited from one site which is a regional genetics centre. In England, the NHS fetal anomaly screening programme ensures that there is equal access to uniform and quality‐assured screening for all pregnant women.

## 4 SOURCES OF UNCERTAINTY IN FETAL GENOME TESTING

All participants recognised an inherent degree of uncertainty in genetic testing *‘that is eternal and always present in genetic diagnosis'* (Dutch 1, clinical geneticist). They acknowledged that technologies which look at the fetal genome increase the diagnostic rate, but at the same time *‘you have more uncertainty'* meaning that ‘w*e diagnose more things, but then in return, when are you sick and when are you healthy? It's no longer black and white'*. (Denmark 4, clinical geneticist).

When asked what the main sources of uncertainty were, the two sources most frequently cited, and which were experienced by participants across all countries, were VUS and IFs. Nevertheless, participants recognised, and many had first‐hand experience of, all sources of uncertainty explored during the interview, highlighting the commonality of those uncertainties in the prenatal setting (Table 2).

**TABLE 2:** Categories and types of uncertainties

Category ‐ uncertainty related to…	Type of uncertainty	Example quote
Incomplete knowledge	Pathogenicity and VUSs	‘It is very often that we find de novo variants, that might be pathogenic, but in a gene that does not have a known significance’. Denmark 2, clinical laboratory geneticist
		‘And the other group would be total novel findings where we find regions that are not—there's not a lot of clinical information about the copy number changes involved, but they're very novel in terms of the data sets that we look at, so they may be quite benign, they might be pathogenic—we're unclear’. Australia 4, clinical scientist
	Gene‐disease correlations	‘We found a deletion of a gene that is very important for the energy conversion inside the brain…but we do not know if this gene can cause illness because we never seen this before’ Denmark 4, geneticist
		‘If you have a gene which is disease associated but the spectrum of mutations does not include large CNVs, that would really be an uncertainty…..if only missense mutations have been found in that gene correlated to disease and then you find a CNV covering the whole gene, that would be uncertain’ Sweden 4, clinical scientist
	How a genetic anomaly presents prenatally	‘So we've had situations where we're just not sure how a condition which we know quite clearly the phenotype, in the postnatal setting, we just don't know how that's going to look on the scan, because there's not enough information about that condition in the prenatal setting’. Singapore 2, paediatrician
		‘I'm well aware from the Page study where there's cases, for example, Sotos syndrome where they presented prenatally with a small head, microcephaly, whereas actually the condition postnatally is associated with the opposite of that, macrocephaly’ UK 6, clinical scientist
		‘There is often no phenotype to hold it up against, or at least a very flimsy one. In week 12 you may know if there is a big nuchal fold. The rest is yet too small for you to see it, so it does make a special challenge in relation to the prenatal’. Denmark 4, geneticist
Unexpected findings	Incidental findings	‘Previously, we had several of those cases where we had a minor ultrasound abnormality and we find Cri‐du‐Chat. I think that's quite common’ Sweden 4, clinical scientist
		‘Recently, I saw a couple with a baby with a 15q11.2 microdeletion, and I mean we know that—and that was just incidental. It was incidentally picked up when they were being evaluated for risk of Turner syndrome. And there was no family history of that’ Singapore 2, paediatrician
		‘I had a case in which we found a de novo variant that caused a fatal disease. It was a foetus that was dead intrauterine and had malformations… But I find out that the foetus is also deaf. It has two variants in the ‘deaf gene’ and both parents are carriers. And then I find out that the mother also has a variant in a large ‘heart gene’, so she may also be carrier of a heart disease’. Denmark 4, geneticist
	Secondary (additional‐looked for) findings	‘Like a BRCA gene—that would not be reported prenatally if it is not asked for. If it is known in the family we would report it, if it is asked’. Sweden 4, clinical scientist
Technology	Technical validity of the result	‘Things that are too small for microarray we won't pick up, yeah, there's promotive variants, so areas around the promotor region of the gene, they're not included in an exome as well. Large indels—so when I'm saying large, I'm saying like over about 30/40 base pairs—the sensitivity of the next generation sequencing because we're using short reads, that also goes down, so there's quite a lot of variants we will miss and we won't get 100% coverage for every single gene as well, so there's a lot we'll still miss’ UK6, clinical scientist
		‘Prenatally you rely on the CVS or amnio, and especially amnio's, we might not get as much DNA. So you might be restricted by how much material you have, and obviously that might lead to suboptimal result. Plus other technical problems like maternal cell contamination and things like that’. UK 1, clinical scientist
		‘We've had samples of poor quality, maternal contamination we can't analyse, haven't met our criteria for quality thresholds. Doesn't happen very often. If possible we rerun the sample or use another technique’. Sweden 5, clinical scientist
	Possible incomplete result	‘If I find a mutation, find a pathogenic mutation in the gene that matches the phenotype, but I don't have a second mutation. Then I will report it anyway, because the second mutation may be somewhere I haven't seen it’. The Netherlands 3, laboratory specialist in clinical genetics
Condition	Incomplete penetrance	‘The other big category of uncertainty we come across is the susceptibility risk, so the autism susceptibility loci, the neuro susceptibility loci where you do see the variant in healthy individuals as well’. Singapore 3, geneticist
		‘Something like a DiGeorge deletion, we have enough evidence on that to know that it's got incomplete penetrance, but also to know that if you see it in a foetus with an abnormal heart, that that is going to be the cause. But then you see it in the next foetus, it doesn't have an abnormal heart. Doesn't mean they're not going to develop one’. UK 2, clinical scientist
	Variable expression variants	‘Even if you've got a known microdeletion, I don't know, something like Phelan‐McDermid syndrome or something like that, there's still quite a wide variation in terms of the degree of learning problems or other things that might crop up like epilepsy and so forth and you can't, you know, it's hard even with a small baby to say “this is what's going to happen”’ UK 4, geneticist
		‘So the same variant can express or in very different ways in different people so we could see a very severe prenatal and it could be severe when they're born, OI is quite a common one where you can see different phenotypes. We've had another one where we had a severe scan for OI and we found mum to be affected and she didn't know she had it but actually when they looked at the clinical history, she'd had quite a few fractures’. UK 6, clinical scientist
Clinical utility	Diagnostic yield	Interviewer: ‘And so what do you think are the main uncertainties in prenatal genomics today?’
		Interviewee: ‘I think that the extent to which like the yield is greater than the testing we currently have in terms of the, the certainty of finding something that would explain a particular, you know, risk or condition’. Australia 1, obstetrician
		‘I usually say that we find something in approximately half of the severely affected ones. The more affected the child or fetus is, the more likely we are to find something. But I have also been dealing with some really difficult children and fetuses, where we did not find the variant’. Denmark 3, clinical geneticist

*Note*: Categories and types of uncertainty taken from a manuscript currently in preparation by Klapwijk et al.

Abbreviation: CNV, copy number variation; VUS, variants of uncertain significance.

## 5 MANAGING UNCERTAINTY

### 5.1 Eligibility for testing

#### 5.1.1 Who should be offered these tests?

There was widespread agreement that these tests should not be offered as a screening test because they are *‘not for reassurance’* and you would *‘pick up stuff you can't explain’* (UK2, clinical scientist). Rather, they should be offered for established indications including: a fetal malformation on ultrasound, an increased risk combined first trimester screening result, a high risk NIPT result and history of a previous pregnancy with a genetic anomaly. It was acknowledged that the criteria for offering ES was different to that of CMA with *‘microarray used for any abnormality, even just increased nuchal translucency…but [use of] prenatal exomes for quite complex multiple abnormalities on the scan’* (UK6, clinical scientist) (Table 3).

Participants frequently spoke in terms of using these tests *‘responsibly’* (UK3, clinical geneticist) and/or to *‘answer a specific question’* (Denmark 4, clinical geneticist). Using the test responsibly was considered important because of the cost of testing in terms of time and resources, and the consequences of detecting VUS and IFs. However, there was evidence that practice differed between countries in relation to testing for parental anxiety. Interviewees from both Sweden and Australia cited this as an example of where testing might be acceptable.

#### 5.1.2 Likelihood of getting a diagnostic result based on indication for testing

Most participants agreed that ES was likely to improve diagnostic yield over CMA due to the higher resolution. The likelihood of getting a result was an important consideration when deciding whether to order a test. Multiple factors could impact the likelihood of getting a result, including: the type and number of fetal anomalies, the quality of the phenotypic data accompanying the sample, and who is reviewing the test results. For ES, the acceptance criteria for testing, whether parental samples are provided, and the expertise of the person triaging the case was also noted as influencing the diagnostic yield. In the case of ES, cases were often reviewed by a clinical geneticist to determine whether ES was appropriate, although for CMA fetal medicine units could order tests.

‘It depends on what your acceptance criteria is…whether a clinical geneticist has looked [and said] “yes I think this is genetics related” or “I think this is another cause”’ UK6, clinical scientist.

### 5.2 Technical limitations of the technology

Interviewees, and in particular clinical scientists, discussed a number of technical challenges that stemmed from methodologies used. These included: the limitations inherent in using ultrasound imaging for phenotyping, particularly in early gestation, where images could be unclear; the limitations of sequencing technology itself, in particular the depth of sequencing (which varies across labs); and the resolution of arrays, resulting in *‘things that are too small for microarray [which] we won't pick up’*. (UK6, clinical scientist). Other potential technical limitations included insufficient DNA to run the test, or maternal cell contamination in the sample. In those cases, a further sample was required which had knock‐on effects for the length of time the parent(s) had to wait for a result, and hence prolonged the uncertainty. Clinical scientists cited examples of a technical limitation whereby a pathogenic variant was found in a recessive gene which fitted the clinical phenotype, but where no second variant was found.

### 5.3 Limitations in current knowledge

#### 5.3.1 Limited information to classify variants

Variant classification was described as an *‘inexact science’* (Australia 3, clinical geneticist), which was subjective, involved *‘interpretation’* (Denmark 3, clinical geneticist)*,* and could result in colleagues interpreting the same result differently. The difficulties of classifying pathogenicity were found to stem primarily from limitations inherent in the systems used to classify variants, notably the lack of available, credible or reliable information where *‘you find one article that says “yes it is associated” and then after five minutes you find in another article in another cohort that says “no, it's not associated”’* (Netherlands 6, clinical geneticist). A number of methodological problems associated with variant interpretation were noted, including small case number and ascertainment bias. One participant articulated that because we are often looking at rare conditions, we *‘don't build up enough data to be very certain’* (UK2, clinical scientist).

In addition, our understanding of the severity of disease‐causing variants may be overestimated as a result of a bias in primarily testing those who are on the more severe end of the spectrum, meaning that ‘*w* *hat* *we'll see in the coming years is we'll start testing patients with milder problems and we might have found that some of them actually have the same variants’*. (Australia 3, clinical geneticist). A geneticist from Singapore noted how much of our understanding is based on European reference datasets and *‘we don't have enough local reference data, genomic datasets’* (Singapore 3, clinical geneticist). Nevertheless, it was hoped that as we do more testing and add to the bank of information, variant classification would improve and *‘something we don't know today, we will know tomorrow’* (Netherlands 3, clinical scientist).

#### 5.3.2 Managing the challenges of variant interpretation

Despite these challenges, participants highlighted various ways of managing the challenges of variant interpretation. These included trio testing, referring to reference databases and filtering tools, and adopting a multidisciplinary approach where clinical scientists and clinicians worked collaboratively. Consulting with colleagues and discussing problematic cases in multidisciplinary team (MDT) meetings occurs across all countries in our dataset, and was considered an important part of results interpretation, for example, *‘it is always collaboration about the interpretation and what we do and do not tell. We confer with each other regularly’*. (Denmark 2, clinical scientist). Participants from the UK and the Netherlands spoke of there being daily or weekly MDT meetings. In addition, they spoke of the option of deferring to an external committee in those cases where *‘the clinician feels they would like an outside opinion’*. (UK3, clinical geneticist).

#### 5.3.3 Limited knowledge of prenatal phenotypes

Participants noted that our current lack of knowledge around natural history in the prenatal setting was a significant challenge for them.

‘A lot of our data from gene function and gene pathology comes from postnatal [samples]…there's things like cardiac and renal [genetic variants that] we won't pick up so much, because we haven't mapped those genes in the prenatal context yet’. UK2, clinical scientist.

Interviewees also gave examples of where fetal phenotypes present differently compared to the postnatal phenotype. For example, a clinical scientist from the UK presented a case of Sotos syndrome where the fetal phenotype presented as prenatal microcephaly, in contrast to the typical postnatal macrocephalic presentation.

## 6 PRACTICAL CHALLENGES

### 6.1 Lack of guidelines

The lack of clinical guidelines for variant interpretation, particularly for the prenatal setting, was a notable practical challenge acknowledged by interviewees. Whilst many cited that they used the ACMG guidelines as a guide, a clinical scientist commented those classification guidelines *‘could be used up to a point, but wouldn't completely apply [in the prenatal context] because postnatally you have a phenotype, prenatally you don't necessarily’* (UK2, clinical scientist). In Denmark, although the Danish Fetal Medicine Society had recently issued guidelines on reporting of variants, one participant noted that *‘it is difficult to make very strict guidelines in a field that moves so fast, so very detailed guidelines are not a good idea’*. (Denmark 5, clinical geneticist).

### 6.2 Fast turn‐around times

A further practical challenge related to the limited timeframe for analysing and returning results in the prenatal setting, which meant that *‘you have to have an opinion…in a relatively short time’* (Netherlands 2, clinical scientist). As a result, participants spoke of their reliance on the tools at their disposal to aid variant interpretation, and thus being limited in their ability to spend time exploring the literature. As one geneticist explained;

‘I have to skate the surface… and I have to rely on the algorithms coded in the computer to find what I need. I don't have time to go back*’*. Denmark 4, clinical geneticist.

However, one participant commented that ES ‘has the potential to give an answer faster than a microarray…sort of breaking that two week boundary’. (Australia 3, clinical geneticist).

### 6.3 Cost

The cost of testing, particularly the cost of ES *‘which is a very expensive test’* (UK6, clinical scientist) compared to CMA, was discussed by some interviewees as being a current limitation of ES over CMA. In Singapore and Australia, patients were required to self‐fund ES, which was *‘a massive barrier’* and meant that *‘only a small minority of public patients would proceed with the test’* (Australia 2, obstetrician). Other costs, such as *‘restructuring the service'* (UK2, clinical scientist) to include prenatal ES, and the potential increased workload for clinical scientists interpreting the data as well as clinicians and genetic counsellors providing lengthier post‐test counselling, was also noted.

## 7 REPORTING

### 7.1 Who decides what to report?

Clinicians and clinical scientists in the UK, Australia and Singapore were in agreement that the clinical scientist makes the final decision as to what results are returned, and whatever is reported on the laboratory report is returned to the parents:

Interviewer: ‘And who decides what results to return and what results not to return?’

*Participant: ‘So pretty much the lab[oratory]. So I guess in this day and age, once something's on the report clinicians would usually return it or at least discuss it’*. Australia 4, clinical scientist.

In Denmark, the Netherlands and Sweden, the clinician makes the final decision, and may not necessarily return all the results provided on the laboratory report.

‘We [clinical scientist] look at the results, write the response that should go to fetal medicine unit, but as soon as it has a pathological response it is checked by a medical doctor. So the reporting is taken over by the medical doctor’. Sweden 3, clinical scientist.

### 7.2 What results are reported?

The criteria for which uncertain results are reported varied both within and across countries. For some types of uncertain results, this was reflective of national guidelines. For other uncertain results, it reflected local practices or was determined on a case‐by‐case basis.

### 7.3 VUS

Reporting practices for VUS varied across countries. In the UK and the Netherlands, reporting practices reflected national reporting guidelines, for example, the Royal College of Pathologists Use of Microarray in Pregnancy, in the UK. In Australia, Denmark, Singapore and Sweden, at the time of interview, there was no formal guidance on whether to report VUS, and so practices differed across laboratories.

‘There aren't really established protocols within Australasia for reporting [VUS] in the prenatal context’. Australia 4, clinical scientist.

In Denmark, The Netherlands and the UK, interviewees did not report VUS in the prenatal setting. In Sweden, Singapore and Australia, clinical scientists stated that they would report VUS if they were of a particular size and in a candidate gene.

‘We report VUS if they are large enough, 1 MB, if its 1 MB and we find some genes that could be of interest that we can't disregard that would be reported as a VUS’. Sweden 4, clinical scientist.

Those interviewees who would not return VUS frequently spoke of wanting to prevent parents from experiencing additional and possibly unnecessary anxiety, or wanting to avoid a situation whereby a patient would terminate a pregnancy on the basis of a VUS result. Where VUS results are returned, interviewees spoke of the need to be *‘honest with patients’* and that *‘paternalism needs to go, even if it's well intentioned’* (Australia 1, obstetrician). In Singapore, the approach was justified on the basis that *‘the patients have paid for the test…most will want to know what all the results are’*. (Singapore 1, obstetrician).

### 7.4 Incidental and secondary findings

Numerous participants were able to cite instances where IFs had been uncovered unintentionally through prenatal CMA/ES. In terms of reporting, there was a general consensus across countries in terms of approach, whereby IFs are reported if they are actionable or clinically significant (to the parent or child): ‘*it's something that could have implications for future pregnancies… if you had a very strong suspicion,* for example *for a cancer gene’* (UK 1, clinical scientist). Late‐onset untreatable conditions are not generally reported, although the exception to this approach was cited by a clinical scientist from Sweden who commented that *‘[the variant] doesn't have to be actionable, just well known’* (Sweden 2, clinical geneticist). Notably, it was highlighted by interviewees from Denmark, the Netherlands and Singapore that patients are usually involved in decision‐making around IFs and are asked to sign a consent form during pretest counselling if they want IFs returned.

None of the participants who took part in the interview study were familiar with a situation whereby secondary findings, disease‐causing genes not related to the primary testing indication but intentionally analysed, were specifically targeted in prenatal CMA/ES. Nevertheless, a clinical scientist from Sweden said that if, for example, there was a known BRCA gene in the family and they were asked to look for it, this was something they would do.

## 8 ISSUES FOR COUNSELLING

Participants frequently mentioned the importance of pretest counselling to manage parent's expectations and posttest counselling to manage the return of uncertain results. A number of recommendations relating to participants' comments are provided in Table 3. There was variability regarding who reported uncertain results to patients. In some countries there was no specific specialist, with participants citing *‘geneticists, genetic counsellors, specialist women's healthcare practitioners, specialist obstetricians’* (Australia 1, obstetrician). In other countries, it was *‘the clinical genetic team’* (UK 6, clinical scientist) or the *‘clinical geneticist, most preferably in collaboration with a fetal medicine specialist’*. (Denmark 5, clinical geneticist).

**TABLE 3:** Managing uncertainty in pre‐ and posttest counselling

Pretest counselling	
Explain the limitations of the technology	*‘One of the things we talk about is the limitations of the test which is that negative result doesn't mean it's not genetic. It could still be genetic it just means the technology has a limitation and we haven't tested everything that we need to test'*. Singapore 3, clinical geneticist
Explain that there is the potential to receive uncertain results including VUS and/or IFs	*‘I explain that prenatally we do not report VUS, that unexpected findings may result. I give examples about the nature of the unexpected findings. It means that all actionable or controllable findings… we report that, because it can have a health benefit if it is found'*. Denmark 1, fetal medicine consultant
	*‘I let them know that if they choose to do the test it does mean if there is a VUS result it will be reported'*. Singapore 4, genetic counsellor
Explain option not to undergo further testing	*‘Present other options including the option of doing nothing which is almost always an option and a very important one. A lot of people feel compelled to do something…so validating that as an option is an important part of the consent process'*. Australia 1, obstetrician
Clarify with patients whether they want to receive uncertain results	*‘I start by addressing how much they want to know'*. Denmark 4, clinical geneticist
**Posttest counselling**	
Tailor the discussion to the patients' needs	*‘I try to tune into the patients' level of understanding and their specific needs'* Denmark 5 clinical geneticist
Highlight what is certain through linking the results from the CMA/ES with what was observed on the ultrasound	*‘If the reason for the examination is structural ultrasound abnormalities, then … you can say something based on the result from the test result. Yes, then I know for sure that the child really has something… it can provide more clarity about what you are talking about'*. Netherlands 1, clinical geneticist
Highlight that many conditions will have been ‘ruled out' through testing	*‘But the moment you have normal results from a particular panel, I say that the chance of an additional problem is not so high any more, because you subsequently excluded many of those disorders'* Netherlands 1, clinical geneticist
Explain that uncertainty will always exist in prenatal testing and the role of human genetic variation	*‘I would frame it in the context of human genetic variation and how common that is and how variable we all are, because I think that as a background piece is very important'*. Australia 3, clinical geneticist
Offer follow‐up appointments to monitor the pregnancy or referring to other specialists	*‘I'd offer them a repeat appointment sooner rather than later if they had further questions or wanted to come back and see me, or appropriate referrals if required, depending on the result'*. Australia 2, obstetrician
Conduct a review once the baby is born to review the baby's progress	*‘If they're continuing a pregnancy, usually I would suggest that they have some sort of review after the baby's born, both to check out the baby make sure things are OK, but also to answer questions'*. Australia 3, clinical geneticist
Signpost parents to support groups or psychological support if available	*‘If they feel overwhelmed with the result and they need to talk to mental wellness service, I tell them the service is readily available for them'*. Singapore 4, genetic counsellor

Abbreviations: CMA, chromosome microarray analysis; ES, exome sequencing;IFs, incidental finding; VUS, variants of uncertain significance

## 9 DISCUSSION

We found that HPs around the globe and from different healthcare systems are dealing with similar *sources* of uncertainty, reinforcing the notion that uncertainties are inherent in the technology and in our limited biological understanding of variants. Notably, however, we identified different strategies to *manage* uncertainty both within and across countries suggesting that the management of uncertainty *is* culturally and/or healthcare‐system specific. A such, global uniformity or consensus is not necessarily a realistic or desirable goal. A similar conclusion was reached by Boormans et al.^12^ who comment that *‘if the most experienced stakeholders (i.e., experts) disagree on what should be detected in prenatal diagnosis, the implementation of a uniform nationwide policy is outdated’*. They suggest that instead, tailor‐made strategies, which incorporate patient's risks and demands and are decided by patients in consultation with their doctor, should be used. This could, for example, include patient‐centred discussions during pretest counselling on the use of higher or lower resolution microarrays, or whether they want to receive VUS or IF results.

There was general consensus on reporting practices for IFs, with HPs reporting those where they would have implications for future pregnancies, but not reporting them if they were adult‐onset conditions. This is reflective of ethical arguments that are prevalent in Western society relating to the importance given to the child's right to an ‘open future’.^37^ Variations were, however, identified when it came to reporting practises for VUS, with some participants only reporting VUS linked to the fetal phenotype and others reporting VUS in candidate genes. These findings echo those of Vears et al.^38^ who found there to be variation in reporting practices of VUS amongst laboratory personnel across Europe, Canada and Australasia. We found that the rationale behind reporting practices for VUS reflected the different values placed on openness (where VUS are reported) versus concerns around causing unnecessary anxiety (where VUS are not reported). These different approaches may reflect differences in national health systems; those with single payer systems may be more conducive to following guidelines, whereas those with more heterogeneous systems and mixed public/private providers may allow for more individualised practices. They may also be linked to cultural differences in how much information parents want and whether there are cultural differences in tolerance for uncertainty; further research here would be valuable.

A further consideration is *how* we define and categorise VUS. For example, 22q11 duplication may be called a VUS in some countries,^9^ and a pathogenic variant of low penetrance or a susceptibility loci in others.^2^, ^39^ The guidelines are also nondirective on this matter; for example, for 22q11 duplication the UK Royal College of Pathologists says *‘Consider detailed scan looking for associated anomalies or reporting in a clinical context’*.^26^ The boundaries that we place on categories and the language that we use is therefore critical if we are to be able to meaningfully discuss and compare approaches. International guidelines may play a role in standardising the way that we categorise variants, although rapidly evolving technology and practice as well as local variation in clinical care and resources, makes international guidelines difficult and likely to become quickly outdated. Nonetheless, insights into the various (best‐)practices of dealing with uncertainty that are often at the core of guidelines, are essential to enable HP's to provide optimal care.

An important finding was the role that HPs play in influencing the management of uncertainty by how they process and discuss it with patients. Parents have expressed that the way uncertain results are communicated has a significant impact on their experience and ability to cope with uncertainty.^40^ Going forwards, it will be important to ensure adequate training for HPs returning CMA and ES results, so that only the more complex cases need to be referred to clinical genetics. Those providing pre‐ and posttest counselling should also have training to support parents in managing uncertainty and make decisions about their pregnancy.^41^ Some attention has been given to how HPs can help manage parental uncertainty in the prenatal setting,^41^, ^42^, ^43^ for example, through emphasising what is known and certain, and through pointing out was is structurally normal.^43^ Further research in this area from the perspective of parents would be valuable. This is particularly important given that there is the potential that uncertain results may impact long‐term maternal psychological outcomes^44^ as well as on the parent/child relationship.^45^

### 9.1 Strengths and limitations

Many of the uncertainties identified in this study mapped onto the Han et al.^32^ taxonomy of uncertainty in genomic sequencing. For example, both studies identified sources of uncertainty including pathogenicity, gene‐phenotype associations, and test limitations as causes of uncertainty. Here, we have validated the taxonomy for the prenatal setting. This is useful in that it supports the use of the taxonomy for education and training purposes amongst health professionals working in prenatal genomics, in particular in thinking about how to ascertain where uncertainties lie and how to evaluate them from a medical standpoint. A limitation of this study was that only a small number of interviews were conducted in each country in a limited number of sites including only one site in the Netherlands and the UK. However, the aim of the study was not to provide a comprehensive overview, but rather provide a snapshot of current practices. Interviews spanned across a year—given the fast pace of change in prenatal genomics, there may have been significant changes during that time, particularly around where different countries are up to in terms of offering ES. At the time of the interview, not all participants had experience of reporting prenatal ES results which may have impacted the findings. Different interviewers conducted interviews in different countries. This may have had an impact on the quality of the interviews conducted; however, we tried to mitigate against this by using a standardised topic guide, and interviews were co‐coded. Finally, due to resource constraints, translations were not back‐translated which is often conducted to check accuracy and quality.^46^ However, the interviewers/translators were all bilingual researchers working in this area and therefore have language and content knowledge to provide technically accurate translations.

## 10 CONCLUSION

Our study highlights the different approaches taken to manage prenatal uncertainty in genomics across differing healthcare settings. A key question raised is whether uniformity in practices is desirable, or indeed, necessary? One value of different healthcare systems adopting differing approaches is the opportunity to compare and contrast the benefits and limitations of the various approaches through both research and meaningful discussion with international colleagues. Further research with parents in different countries to compare their preferences towards and experiences of receiving uncertain findings would also add to our understanding in this area. Whilst uncertainty in prenatal genomics clearly raises a myriad of challenges, we should not be too disheartened; these new genomic technologies are transforming healthcare and offering new opportunities for prospective parents to make important choices about their pregnancy.

## Data Availability

Qualitative datasets generated during the current study are available from the corresponding author on reasonable request.
